# The STOP Program: a Hybrid Smoking Prevention and Cessation Training for Cancer Care Providers in Colombia and Peru

**DOI:** 10.1007/s13187-023-02322-8

**Published:** 2023-06-27

**Authors:** Irene Tamí-Maury, Samuel Tundealao, Vilma Díaz, Elizabeth Ochoa, Esperanza Garcia, Johanna Rincon, Valeri Noé-Díaz, Carlos Castañeda, Jesús Acosta, María Fernández, Tatiana Vidaurre, John Crowley

**Affiliations:** 1 Department of Epidemiology, Human Genetics, and Environmental Sciences, School of Public Health, The University of Texas Health Science Center at Houston, 1200 Pressler Street, Suite E641, Houston, TX 77030 USA; 2 Instituto Nacional de Enfermedades Neoplasicas (INEN), Lima, Perú; 3 Instituto Nacional de Cancerología (INC), Bogotá, DC Colombia; 4 Universidad Intercontinental (UIC) in Mexico City, Mexico City, Mexico; 5 Department of Health Promotion & Behavioral Sciences, School of Public Health, The University of Texas Health Science Center at Houston, Houston, TX USA; 6 Cancer Research and Biostatistics (CRAB), Seattle, WA USA

**Keywords:** Cancer care providers, Cigarette, Smoking prevention and cessation, Latin America, Training program

## Abstract

We designed and tested the feasibility of the Smoking Cessation Training Program for Oncology Practice (STOP), a hybrid (face-to-face plus web-based) educational intervention to enhance Spanish-speaking cancer care professionals’ (CCPs’) ability to provide brief smoking prevention and cessation counseling to cancer patients and survivors. Changes in the CCPs’ competencies (knowledge, attitude, self-efficacy, and practices toward smoking and smoking cessation services) were assessed post-training. Sixty CCPs from one major cancer center in Colombia (*n* = 30) and Peru (*n* = 30) were invited to participate in a 4-module hybrid training program on smoking prevention and cessation. Demographic and pre- and post-test evaluation data were collected. The training’s acceptability was measured after each module. Bivariate analysis was conducted using Wilcoxon signed-rank test to compare the CCPs’ competencies before and after the delivery of the STOP Program. Effect sizes were computed over time to assess the sustainability of the acquired competencies. Twenty-nine CCPs in Colombia and 24 CCPs in Peru completed the STOP Program (96.6% and 80.0% retention rates, respectively). In both countries, 98.2% of the CCPs reported that the overall structure and organization of the program provided an excellent learning experience. The pre-post-test evaluations indicated that the CCPs significantly improved their knowledge, attitude, self-efficacy, and practices toward smoking, smoking prevention, and cessation services. We found that the CCPs’ self-efficacy and practices increased over time (1-, 3-, and 6-month assessments after completing the 4 educational modules). The STOP Program was effective and well-received, demonstrating remarkable changes in CCPs’ competencies in providing smoking prevention and cessation services to cancer patients.

## Introduction

Smoking prevention and cessation are one of the most effective ways to prevent cancer and is also essential after a cancer diagnosis to improve treatment outcomes [1]. Continued smoking after a cancer diagnosis has been linked with shorter survival, recurrent disease, decreased treatment efficacy, and reduced quality of life [1]. In the United States (US) and other high-income countries, a cancer diagnosis has been used as a window of opportunity to intervene and provide smoking prevention and cessation assistance to patients and survivors [2, 3]. Findings from a cross-sectional survey conducted among 238 cancer care providers (CCPs) in Colombia and Mexico showed that lack of training in smoking prevention and cessation counseling was among the most important barriers to providing cessation services in oncology settings [4]. This preliminary study indicated the need to enhance the capacity of CCPs to effectively deliver smoking prevention and cessation counseling to their patients and integrate such services into cancer care with the ultimate goal of reducing cancer recurrence, treatment-related complications, and mortality among Latin American (LA) patients.

The use of information technologies to deliver online educational programs and the widespread use of mobile health technology (mHealth) are allowing for innovative ways to improve healthcare delivery [5]. Few smoking cessation programs for healthcare professionals (HCPs) in LA have been reported in the scientific literature [6–10]. The Fruitful study, a 2016 smoking cessation training program for HCPs from Bolivia, Guatemala, and Paraguay based on the 5A’s model of smoking cessation, reported that the participants had statistically significantly higher performance scores in each 5A’s strategies 6 months after receiving the training [8]. A 2016 report from 79 different care centers in Mexico which participated in smoking cessation training between 2012 and 2014 showed that health professionals from 93.6% of those centers regularly provide brief counseling services to their patients [9]. To our knowledge, there are limited culturally appropriate training programs in Spanish specifically designed for CCPs in LA to build their capacity on smoking prevention and cessation.

Smoking cessation can improve the overall health and quality of life of cancer patients, as well as reduce the risk of dying from cancer [1]. Thus, prompt identification of smoking behavior and delivery of interventions are critical for improving cancer treatment. Delivering tailored smoking prevention and cessation interventions for cancer patients can be challenging, particularly in LA, where there is a lack of trained cancer clinicians and/or limited referrals to smoking treatment services. A cost-effective solution in LA oncology settings is to train CCPs on how to provide brief smoking prevention and cessation counseling services that respond to the needs of cancer patients in the region.

Therefore, in partnership with two major LA cancer centers located in Colombia and Peru, we developed, implemented, and evaluated STOP (Smoking Cessation Training for Oncology Practice) Program, a smoking prevention and cessation hybrid training program specifically designed for Spanish-speaking CCPs to safely and effectively provide brief smoking prevention and cessation counseling to cancer patients and survivors. In addition, we assessed the difference in the competencies (smoking cessation knowledge, attitudes, self-efficacy, and practices) among the CCPs before and after their participation in the training program.

## Methods

### Cancer Centers

In 2018, our team partnered with the National Cancer Institute (INC) in Colombia and the National Institute of Neoplastic Diseases (INEN) in Peru, two of the leading cancer centers in LA, to design, implement, and deliver the STOP Program.

### Targeted Audience

A convenience sample of sixty targeted CCPs (30 in each country) was invited to participate in the program. The inclusion criteria were (1) CCP working at one of the above centers; (2) ≥ 18 years old; (3) had direct contact with cancer patients; and (4) proficiency/access to internet with a computer, tablet, or smartphone.

### Ethical Statement

INC in Colombia (Cod INC-STOP Protocol/EX- 00812), the University of Texas MD Anderson Cancer Center Houston (Protocol No. PA17-0878), and the University of Texas Health Science Center Houston (Protocol No. HSC-SPH-20–1339) approved the protocol for the program. Protocol approval was not needed from the INEN in Peru as this was an educational program. Informed consent was obtained from all participating CCPs.

### Training Curriculum

The STOP Program, a hybrid (face-to-face plus web-based) training focused on different aspects of smoking prevention and cessation, was culturally and linguistically developed for LA oncology settings. The program consisted of 4 educational modules delivered within a month. Each module lasted 1 h, and after its delivery, STOP trainees were invited to complete a post-module evaluation to assess the module-content acceptability.

The modules were as follows:

- Module I: historical overview and epidemiology of tobacco use in LA
- Module II: cancer and other chronic conditions associated with smoking
- Module III: behavioral-based smoking prevention and cessation interventions
- Module IV: cessation medications and therapies

Modules I, III, and IV were presented online, while Module II was in person. Once the 4-module course was delivered, trainees were invited to participate in 6 video conferencing sessions (one per month) where their experiences and challenges in providing smoking cessation services to their patients were discussed.

### Data Collection

The STOP Program was delivered between April and October 2018 in Peru and January and July 2019 in Colombia. A pre-post-design was used to evaluate the STOP Program. There were four online assessments delivered at the following time points:

- T0 = baseline (before the hybrid course)
- T1 = 1 month (immediately after the hybrid course)
- T3 = 3 months (mid-point assessment of STOP training program)
- T6 = 6 months (final assessment of STOP training program)

### Measures

The domains and questions included in the online assessments were adapted from the International Association for the Study of Lung Cancer Survey and the National Health Interview Survey [11, 12]. REDCap, a secure web-based application, was used for building and managing online research surveys. There were 16 questions in knowledge domain, 6 in attitude domain, 7 in self-efficacy domain, and 7 in practice domain. These items were included in the online T0, T1, T3, and T6 assessments. Sociodemographics (age, gender, etc.) and self-reported smoking behavior information were collected from each CCP at baseline (T0). The STOP training was the exposure while the CCPs’ acceptability of the program, changes in the CCPs’ knowledge, attitude, self-efficacy, and practices toward smoking and smoking cessation services were the outcomes.

### Statistical Analysis

The retention rate was used as a key parameter for assessing the feasibility of the STOP Program. The retention rate at each training site was the proportion of recruited CCPs who remained in the training program and completed the 6-month assessment (T6). Descriptive statistics were used to characterize the sociodemographic information collected at T0. The responses to the acceptability questions were dichotomized, with proportions computed for each educational module.

Gains in participants’ competencies in providing smoking prevention and cessation services to cancer patients were assessed by comparing responses to selected domain questions at each of the 4 assessment times. Each correct knowledge question response was assigned a score of 1; the incorrect answer was assigned 0. Attitude, self-efficacy, and practice domains had questions with responses on a 5-point Likert scale. Possible score ranges were 0–16 (knowledge), 6–30 (attitude and practice), and 7–35 (self-efficacy). The Wilcoxon signed-rank test was used to compare the scores obtained in the pre-test and post-test of the training program. Effect sizes were computed between the post-test (T1, T3, and T6) and baseline (T0) scores to assess the sustainability of the acquired competencies. Data analysis was conducted with STATA 17.0/SE.

## Results

### Retention Rate

In Colombia, the post-test retention rate was 96.7%, whereas in Peru, it was 80.0%. The overall retention rate was 88.3%.

### Sociodemographic Characteristics

The average age of the CCPs was 36 (± 8.5) years. There were 54 female CCPs and 6 male CCPs. All Peruvian CCPs were oncology nurses, while all except two of the Colombian CCPs were nurses. About 40% of the CCPs were specialists or had a master’s or doctorate degree. None of the CCPs were current smokers. However, 8.3% were former smokers. Most CCPs (83.3%) devoted more than 50% of their clinical time to patient care (Table 1).

**Table 1:** Sociodemographic characteristics of the CCPs at T0 (pre-test)

	Total (*n* = 60)	Colombian CCPs (*n* = 30)	Peruvian CCPs (*n* = 30)	*p*-value
Age				
Mean (SD) years	35.7 (± 8.48)	35.0 (± 8.78)	36.3 (± 8.28)	0.577^a^
Gender				**0.024**^b^
Male	6 (10.0%)	6 (20.0%)	0 (0.0%)	
Female	54 (90.0%)	24 (80.0%)	30 (100.0%)	
Highest level of education				0.114^c^
College	36 (60.0%)	15 (50.0%)	21 (70.0%)	
Post-college, such as specialization, master’s, PhD	24 (40.0%)	15 (50.0%)	9 (30.0%)	
Profession				0.980^b^
Nurses	58 (96.7%)	28 (93.3%)	30 (100.0%)	
Physicians	2 (3.3%)	2 (6.7%)	0 (0.0%)	
Years since the last educational degree				0.321^c^
Less than 5 years	33 (55.0%)	18 (60.0%)	15 (50.0%)	
5–10 years	15 (25.0%)	5 (16.7%)	10 (33.3%)	
Greater than 10 years	12 (20.0%)	7 (23.3%)	5 (16.7%)	
Smoking status				0.640^b^
Current smokers	-	-	-	
Former smokers	5 (8.3%)	2 (6.7%)	3 (10.0%)	
Never smokers	55 (91.7%)	28 (93.3%)	27 (90.0%)	
Time devoted to patient care				0.171^b^
< 50%	10 (16.7%)	3 (10.0%)	7 (23.3%)	
50–74%	30 (50.0%)	14 (46.7%)	16 (53.3%)	
75–100%	20 (33.3%)	13 (43.3%)	7 (23.3%)	

Bold value indicates statistical significance (*p* < 0.05)

^a^*p*-value from the *t*-test

^b^*p*-value from the Fisher exact test

^c^*p*-value from the chi-square test

### Training Acceptability

Most CCPs (> 98%) said that each module’s content was pertinent and informative. More than 96% of respondents said they would implement the modules’ teachings into their practice. More than 88% of the CCPs found the earlier readings, additional material, discussions, and group activities to be of immense help. The time allotted for the presentation, questions, responses, discussions, and group activities was deemed adequate by more than 90% of the CCPs. More than 98.2% of the CCPs concluded that the program’s overall organization provided an excellent learning experience.

### Changes in Competencies

Overall, the comparisons between T0, T1, T3, and T6 assessments indicated that both cohorts participating in the STOP Program improved their knowledge, attitude, self-efficacy, and practices toward cigarette smoking and smoking cessation (Table 2). Immediately after completing the 1-month course (T1), Colombian and Peruvian CCPs reported higher scores for knowledge (*p* = 0.0001), positive attitude toward smoking cessation (*p* = 0.0008), increased perceived self-efficacy to offer smoking prevention and cessation services to cancer patients (*p* < 0.0001), and engagement in smoking prevention and cessation practices for their patients compared to T0 (Table 2).

**Table 2:** Changes in knowledge, attitude, self-efficacy, and practices between baseline (T0) and 1-month assessment (T1)

	Colombia and Peru			Colombia			Peru		
	Pre-test (T0)	Post-test (T1)	*p*-value	Pre-test (T0)	Post-test (T1)	*p*-value	Pre-test (T0)	Post-test (T1)	*p*-value
Knowledge									
Mean (SD)	10.8 (± 2.39)	12.3 (± 1.94)	*0.0001*^*c*^	10.6 (± 2.55)	12.6 (± 1.97)	*0.0004*^*c*^	11.0 (± 2.24)	12.1 (± 1.97)	*0.0012*^*c*^
Attitude									
Mean (SD)	32.3 (± 2.86)	33.4 (± 2.14)	*0.0008*^*c*^	32.1 (± 3.21)	33.4 (± 2.06)	*0.0206*^*c*^	32.5 (± 2.54)	33.5 (± 2.30)	*0.0138*^*c*^
Self-efficacy									
Mean (SD)	23.5 (± 5.67)	27.4 (± 5.05)	< *0.0001*^*c*^	22.7 (± 5.49)	27.5 (± 4.49)	< *0.0001*^*c*^	24.3 (± 5.81)	27.3 (± 5.79)	*0.0079*^*c*^
Practice									
Mean (SD)	20.5 (± 5.15)	24.3 (± 4.45)	*0.0001c*	19.3 (± 5.35)	23.8 (± 4.75)	*0.0012*^*c*^	22.0 (± 4.64)	25.1 (± 4.75)	*0.0420*^*c*^

Italic values indicate statistical significance (*p* < 0.05)

^c^*p*-value from the Wilcoxon signed-rank test

### Competency Sustainability

The training’s effect sizes on knowledge and attitude declined at T3 but then increased at T6. An increasing trend in the training’s effect size on CCPs’ smoking prevention and cessation service self-efficacy and practices from T1 was observed in T3 and T6 (Table 3).

**Table 3:** Effect size of the training program on knowledge, attitude, self-efficacy, and practices at T1, T3, and T6

	Colombia and Peru			Colombia			Peru		
	T1	T3	T6	T1	T3	T6	T1	T3	T6
Knowledge	0.7	0.5	0.7	0.9	0.5	0.8	0.5	0.4	0.5
Attitude	0.5	0.1	0.2	0.5	0.1	0.2	0.4	0.5	0.4
Self-efficacy	0.7	0.7	1.1	1.0	0.8	1.5	0.5	0.6	0.8
Practice	0.8	1.4	1.8	0.9	1.7	2.2	0.7	1.1	1.5

## Discussion

The majority of smoking prevention and cessation training programs are only available in English and implemented in developed and high-income countries [13–16]. Only a few of these programs are specifically designed for providers caring for cancer patients. Generally speaking, most low- and middle-income countries (LMIC), including some LA countries, undertake smoking prevention and cessation initiatives ineffectively and/or inconsistently in oncology settings [17]. Due to a lack of personnel and necessary resources, LA cancer centers mainly concentrate on cancer diagnosis and treatment, dismissing partially or entirely preventive efforts, such as smoking prevention and cessation counseling, that can help cancer patients’ treatment outcomes, improve their quality of life, and increase their overall survival rates [17]. To the best of our knowledge, the STOP Program is the first formal smoking prevention and cessation training effort specifically developed for Spanish-speaking CCPs in LA.

The STOP Program recorded a retention rate of 96.7% in Colombia and 80% in Peru (combined retention rate was 88.3%). These outstanding rates set it apart from other training programs [8, 13, 18]. Although the Fruitful study’s recruitment rate was not reported, 99 out of 202 participants completed the post-test training/evaluations at 6 months, resulting in 49% retention rate. The retention rate for the STOP Program was approximately twice higher than for the Fruitful study [8].

The STOP Program is culturally appropriate to LA, a region that values some form of face-to-face interaction despite the tremendous growth of online communication. The training was also culturally adapted because the educational contents were LA-specific and country-specific. We believe this hybrid training program could be more cost-effective and advantageous than in-person or online training only. The STOP Program can offer a convenient alternative to receiving training on smoking prevention and cessation with little disruption to CCPs’ hectic clinical schedules providing care to cancer patients. In the program, more than 98% of the CCPs acknowledged and commended the training program’s overall organization as an excellent learning platform and said they would suggest the training to their other cancer provider colleagues. This further reinforces the high feasibility of the STOP Program’ success among CCPs in LA countries. The format and content of the program were highly accepted by the participating CCPs.

The STOP Program was conceived and designed with the idea of increasing the engagement of the CCPs in prevention and cessation counseling for cancer patients. In addition to the module’s content and strategically selecting the speakers, there were post-training touch-base sessions to discuss the CCPs’ challenges and experiences while applying the knowledge they acquired to their daily clinical activities. These monthly touch-base sessions after T1 allowed for a two-way learning process in which participants shared with the rest of the cohort trainees their challenges in providing smoking prevention and cessation services and attempted to develop solutions. Other training programs in the region did not have such follow-up sessions with the participants after receiving the educational content, missing an opportunity to know the challenges the professionals encountered when attempting to apply the acquired knowledge to their clinical work.

Our findings demonstrated that the STOP Program significantly improved the competencies of the participating CCPs in terms of knowledge, attitude, self-efficacy, and practices. Like the brief smoking prevention and cessation training effort among HCPs in Malta [19], the STOP Program significantly increased smoking and smoking cessation knowledge among CCPs in Peru and Colombia. In line with the Tobacco Treatment Training-Oncology (TTT-O) study among CCPs in the USA [20], the Fruitful study among HCPs in LA [8], and another study among HCPs in the USA [21] by Payne et al., there was a significant increase in the CCPs’ self-efficacy in providing smoking prevention and cessation services, signifying increased motivation to provide smoking prevention and cessation services to their cancer patients. Payne et al. found almost no change in the negative attitudes of the HCPs after training [21]. By contrast, the STOP Program reported an increase in the perceived positive attitude of the CCPs toward the importance of smoking prevention and cessation in cancer care. Like the previous reports [8, 14, 19, 21], the practice domain in the STOP Program recorded one of the highest significant increases post-training. Our findings showed that the STOP Program significantly improved smoking prevention and cessation efforts and practices like the other reported smoking cessation training programs in the literature. In addition to current knowledge on the pre-post-beneficial effects of smoking prevention and cessation training programs on HCPs, our training program goes a step further to demonstrate sustainability in the competencies acquired during the training. We found an increasing trend in the training’s effect size on CCPs’ smoking prevention and cessation service self-efficacy and practices at one, three, and 6 months after training.

There were some limitations to this training effort. To begin, the STOP Program was tested among a small sample of CCPs from only two LA countries, undermining the generalizability of our findings for the LA region. Second, the evaluation of the program was self-reported, which could have prompted response bias. Although all the online sessions went seamlessly, the internet services in Colombia and Peru constituted some technical difficulties in connecting and having all the equipment ready for the online meetings. Lastly, a third cohort of the STOP Program was planned in Brazil. However, due to the COVID-19 epidemic, the training was not fully implemented.

Despite these limitations, the STOP training program is one of the first educational efforts to be developed and evaluated among CCPs in the LA region, with results demonstrating high feasibility and remarkable change in the CCPs’ competencies in providing smoking prevention and cessation services to cancer patients.

## Conclusion

The STOP Program was both effective and well-received. Its implementation showed sustained beneficial effects among CCPS in the oncology environment beyond the simple acquisition of tobacco use and nicotine addiction knowledge, with significant competency increase in providing smoking prevention and cessation services to cancer patients. Although preliminary, these results provide a justification for continued development and evaluation of this hybrid training program in other LA oncology settings.

## Data Availability

Data is available upon request.

## Declarations

### Disclaimer

The sponsor did not influence in any way in the design, the data collection, the analysis, the writing, and the decision to publish these results.
